# Comparison of CT and Dixon MR Abdominal Adipose Tissue Quantification Using a Unified Computer-Assisted Software Framework

**DOI:** 10.3390/tomography9030085

**Published:** 2023-05-20

**Authors:** Li-Yueh Hsu, Zara Ali, Hadi Bagheri, Fahimul Huda, Bernadette A. Redd, Elizabeth C. Jones

**Affiliations:** Department of Radiology and Imaging Sciences, Clinical Center, National Institutes of Health, Building 10, Room 1C370, 10 Center Drive, Bethesda, MA 20892, USA

**Keywords:** abdominal adipose tissue, computed tomography, magnetic resonance imaging, fat quantification, image segmentation

## Abstract

Purpose: Reliable and objective measures of abdominal fat distribution across imaging modalities are essential for various clinical and research scenarios, such as assessing cardiometabolic disease risk due to obesity. We aimed to compare quantitative measures of subcutaneous (SAT) and visceral (VAT) adipose tissues in the abdomen between computed tomography (CT) and Dixon-based magnetic resonance (MR) images using a unified computer-assisted software framework. Materials and Methods: This study included 21 subjects who underwent abdominal CT and Dixon MR imaging on the same day. For each subject, two matched axial CT and fat-only MR images at the L2-L3 and the L4-L5 intervertebral levels were selected for fat quantification. For each image, an outer and an inner abdominal wall regions as well as SAT and VAT pixel masks were automatically generated by our software. The computer-generated results were then inspected and corrected by an expert reader. Results: There were excellent agreements for both abdominal wall segmentation and adipose tissue quantification between matched CT and MR images. Pearson coefficients were 0.97 for both outer and inner region segmentation, 0.99 for SAT, and 0.97 for VAT quantification. Bland–Altman analyses indicated minimum biases in all comparisons. Conclusion: We showed that abdominal adipose tissue can be reliably quantified from both CT and Dixon MR images using a unified computer-assisted software framework. This flexible framework has a simple-to-use workflow to measure SAT and VAT from both modalities to support various clinical research applications.

## 1. Introduction

Obesity is a growing global health problem, with emerging evidence suggesting that accumulation of abdominal fat is more strongly associated with health risks such as type 2 diabetes mellitus, atherosclerosis, and hypertension [1]. Intraabdominal fat is composed of both subcutaneous adipose tissue (SAT) and visceral adipose tissue (VAT). VAT, in particular, is a risk factor for the development of clinically significant metabolic syndrome [2] and cardiometabolic disease [3]. Measuring waist circumference is a rapid and easy method to obtain an estimate of the abdominal adipose tissues, but it does not accurately differentiate SAT from VAT. For more accurate quantification and assessment of abdominal adipose tissue distribution, the standard method for fat distribution analysis is by cross-sectional imaging such as computed tomography (CT) and/or magnetic resonance imaging (MRI). A robust software tool that allows for accurate assessment of SAT and VAT on either CT or MR imaging would be clinically beneficial because of its flexibility and potential cost savings.

Abdominal CT imaging can acquire high-resolution images with less motion artifact than MRI due to a very rapid scan time [4]. CT uses a standardized Hounsfield Unit (HU) to express the attenuation differences of tissues that correspond to inherent tissue characteristics, allowing for accurate identification of SAT and VAT. CT imaging is the clinically preferred method of adipose tissue quantification, but the risk from ionizing radiation exposure makes CT suboptimal for serial measurements, especially in children. MRI does not use ionizing radiation, but MRI tissue signal characteristics, unlike computed tomography (CT), depend on both tissue properties and acquisition protocol and can therefore vary from scan to scan. MRI does not have standardized units to measure signal intensity levels for different tissues and relies on arbitrary units that remain susceptible to magnetic field inhomogeneities [4,5]. Among various MR imaging techniques, the multipoint Dixon-based fat and water separation technique has emerged as an effective approach for the assessment of adipose tissue because a fat-only image can be generated to provide excellent signal intensity contrast, highlighting adipose tissue in different abdominal compartments [6,7].

To quantify abdominal adipose tissues and to differentiate SAT versus VAT, segmentation of abdominal wall muscle layers from the axial images is often required. While manual segmentation by experienced readers has been conducted in previous studies [8,9,10,11], it is a tedious and time-consuming process that is impractical for large image datasets. Thus, there is a continued demand for computer-assisted automated methods that are easy to use and can quickly and accurately segment and quantify abdominal adipose tissue on both CT and MR images.

Several approaches have been proposed to computerize the quantification of SAT and VAT from either CT or MR abdominal images [5,12]. However, none of these methods have been shown to be able to automatically quantify SAT and VAT from both imaging modalities. Furthermore, while both CT and MR imaging are commonly used to measure adipose tissue in the abdomen, few studies have provided a cross-modality comparison of SAT and VAT from the same individuals scanned on the same day.

In this study, we aimed to compare quantitative measures of abdominal adipose tissue in the same subjects who underwent same-day CT and MR imaging. We presented a universal computer-assisted software framework that can quantify SAT and VAT from both CT and Dixon MR images. This software platform provides the flexibility, ease of use, and simple workflow integration for routine abdominal adipose tissue quantification from CT and MR images interchangeably and facilitates internal comparison between these two modalities.

## 2. Materials and Methods

### 2.1. Study Subjects

This study was conducted under quality improvement of the Clinical Image Processing Service within the Radiology and Imaging Sciences department of the National Institutes of Health (NIH) Clinical Center, which was exempted from informed consent by the institutional review board. Between April 2021 and 21 July 2021, subjects who underwent both CT and MR abdominal imaging on the same day were included in this study prospectively to evaluate an in-house-developed computer software for abdominal fat quantification.

### 2.2. Image Acquisition

Both CT and MR image acquisition were performed at NIH Clinical Center Radiology Imaging and Sciences. All abdominal CT images were acquired using a Siemens SOMATOM scanner (Siemens Healthineers, Erlangen, Germany) with typical scanning parameters of 90–120 kVp tube voltage with CARE Dose automatic exposure control enabled, 2 mm axial slice thickness, field of view 384 × 381 mm^2^–500 × 500 mm^2^, acquisition matrix size of 512 × 512, and at feet-first prone position.

All abdominal MR scans were acquired on a 1.5T Siemens MAGNETOM scanner (Siemens Healthineers, Erlangen, Germany) using standard three-dimensional two-point Dixon T1 weighted imaging sequence with typical acquisition parameters of 6.71 ms repetition time, 1.19 ms and 2.39 ms echo time, 10^◦^ flip angle, 475 Hz/pixel bandwidth, 81.25 percent phase field of view, 3 mm slice thickness, 365 mm × 450 mm field of view, 320 × 195 acquisition matrix, and 320 × 260 reconstruction matrix. Two axial image series were acquired to cover L2–L3 and L4–L5 spine segments separately.

### 2.3. Image Processing

De-identified CT and MR images were requested from the NIH Biomedical Translational Research Information System and retrieved from a Carestream Vue Picture Archiving and Communication System (PACS, Carestream Health, Rochester, NY, USA). For each subject, an experienced radiologist selected two matched CT and MR image pairs, one at the level of L2–L3 and the other at the level of L4–L5 lumbar disc spaces, using Vue Desktop Diagnostic Client (version 12.2). A total of 42 pairs of images were selected and analyzed. For the MR scans, fat-only Dixon images were used for processing and comparison.

**Figure 1:**
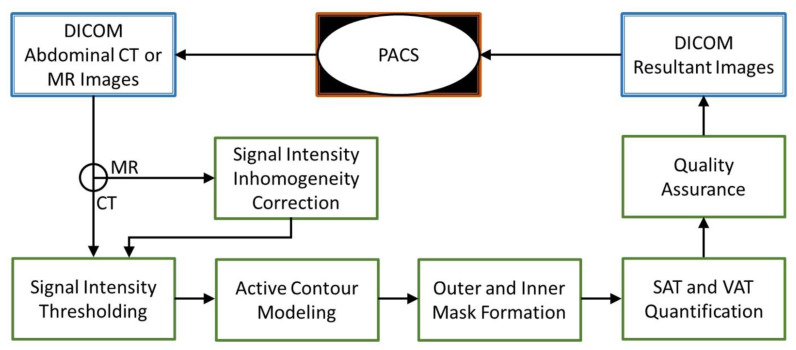
Image processing pipeline for the proposed computer-assisted approach for CT and MR abdominal adipose tissue quantification.

In-house software was developed and used for processing, segmentation, and measurement of abdominal adipose tissue compartments for both CT and MR images. The software was developed in Interactive Data Language (IDL, Harris Geospatial Solutions, Melbourne, FL, USA) with DCM4CHE Java-based programming library for Digital Imaging and Communications in Medicine (DICOM) networking and file handling. An outline of the proposed software system is shown in Figure 1. This framework is similar to the previous methods [13,14] but developed in an independent software platform and with separate image processing functions. To facilitate a practical workflow for routine clinical image processing, our software also incorporates an integrated graphical user interface to improve image segmentation and quantification accuracy, as well as DICOM network functionality for image transmission and storage.

After loading the image, the software automatically generates an outer body mask and an inner body mask to delineate the abdominal wall muscle layer based on signal intensity thresholding where −190 HU was used for the CT, and 50% of the max intensity was used for the MR. For MR images, a separated processing step for correcting B_1_-field-related signal inhomogeneity was implemented before thresholding [15]. Next, the outer and inner abdominal wall tracing was performed based on an active contour model, or snake, [16] using initial body masks from region growing. The snake is an energy-minimizing spline steered by external constraint and image forces that pull it toward nearby edges. The energy of the snake is calculated from its shape and location within the image. The snake method has been proved to be an effective method in contour detection, and it has been employed in many medical image applications [17]. On each MR and CT image, an outer contour around the abdominal wall was detected by shrinking the convex hull of the external body mask. An inner contour was then detected by shrinking the outer contour further toward the internal abdominal wall. These contours were generated automatically without any user input.

For fat quantification on the CT images, an intensity window of −190 to −45 HU was selected to measure the total adipose tissue area (in cm^2^) in each slice. On the MR images, a signal intensity threshold at 50% of the maximum intensity range was used to calculate the total adipose tissue area (in cm^2^) in each slice. The SAT area was calculated by summing the total adipose tissue pixels between the outer and inner contour space. The VAT area was calculated by summing the total adipose tissue pixels within the inner contour space. After the automatic contour detection and fat quantification, the user can adjust the contours, as well as modify the adipose pixel masks, using built-in contour and mask editing tools in the software.

Both CT and MR images were processed independently and blindly. After the automated abdominal wall segmentation and adipose tissue quantification, the computer-generated results were quality checked by an experienced radiologist with 28 years of experience (HB) and edited if necessary to remove false positive pixels. Areas of fat inside the spinal canal and within the neural foramina as well as intramuscular fat in the paravertebral muscles were considered as unwanted false positive adipose pixels. The resultant images and masks were saved in secondary capture DICOM format and ready to be archived in the PACS.

### 2.4. Statistical Analysis

Outer and inner abdominal region segmentation as well as VAT and SAT pixel classification were evaluated separately with results of MR compared against CT. Results were recorded as mean ± standard deviation (SD). Agreement between the CT and MR measured results was evaluated using Pearson correlation coefficient and Bland–Altman plot. A Kolmogorov–Smirnov test was used to assess the normality of the variables using SPSS statistical software (IBM Corp., Armonk, NY, USA). A two-tailed, paired Student’s t-test was used to determine statistical significance. A *p*-value < 0.05 was considered statistically significant for each test.

## 3. Results

There were 12 male and 9 female subjects included in the study. The mean length of time between CT and MR imaging was 2.8 ± 2.3 h (range 0.6–8.0 h), and 8 CT scans were performed before MRI. The mean age was 56 ± 14 years (range 31–78 years). The mean body mass index (BMI) was 27.5 ± 4.6 kg/m^2^ (range 18.7–37.9 kg·m^−2^). Table 1 summarizes the demographic information for the study participants.

The automatic computer segmentation time per image was less than a second for CT and two seconds for MR images. The software framework automatically generated a set of contours to delineate inner and outer abdominal wall, as well as two color masks to depict SAT and VAT pixels. The time for manual editing varies from a few seconds to a minute or two for removing false positive fat pixels and correcting body contours if needed.

**Figure 2:**
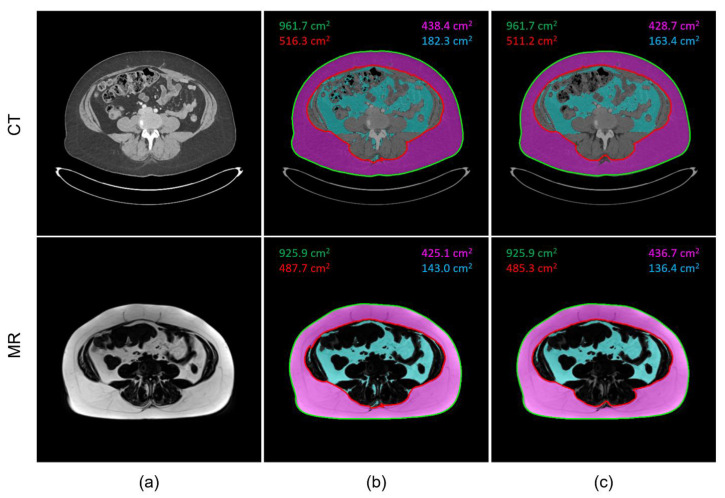
Qualitative comparison of CT (top row) and MR (bottom row) image segmentation and fat quantification based on the proposed approach. (**a**) An example matched image pair at the level of L4–L5 vertebrae. (**b**) Computer automatically generated abdominal wall boundary and adipose tissue masks, where the outer contour is displayed in green and the inner contour in red; SAT and VAT regions are shown in magenta and cyan color masks, respectively. (**c**) The outer and inner contours as well as SAT and VAT masks after the manual corrections.

Figure 2 provides an example of the segmentation results for the inner and outer abdominal cavity, as well as the quantified SAT and VAT areas. A matched CT and MR image pair of the same patient is shown in Figure 2a. The computer automatically generated abdominal wall boundaries and adipose tissue masks on both images are shown in Figure 2b. The outer and inner body contours as well as SAT and VAT pixel masks after manual corrections are shown in Figure 2c. Typical false positive fat pixels include intramuscular fat within paravertebral muscles, small islands of fat in the neural foramina and spinal canal on the MR images, as well as intracolonic contents on the CT images, since these pixels could share similar signal intensities with SAT and VAT.

For quantitative comparison of CT versus MR image segmentation and fat quantification, Table 2 summarizes the mean, standard deviation, and the range of the outer and inner abdominal regions as well as the SAT and VAT areas. To assess the agreement between CT and MR measurements, Pearson coefficients were 0.97 for both outer and inner region segmentation, 0.99 for SAT, and 0.97 for VAT quantification (all *p* < 0.01). Bland–Altman analyses showed there were minimum biases of −1.90 cm^2^, −1.51 cm^2^, −0.13 cm^2^, and −1.96 cm^2^ for outer, inner, SAT, and VAT areas comparing MR against CT measurements. Furthermore, the normality test using the Kolmogorov–Smirnov test shows that all variables follow a normal distribution (all *p* > 0.05). Student’s t-test shows the difference between CT and MR measurements is not statistically significant for comparing outer (*p* = 0.59) and inner (*p* = 0.58) regions, as well as SAT (*p* = 0.96) and VAT (*p* = 0.26) quantification. Overall, our results showed there were excellent agreements for SAT, VAT, and abdominal cavity areas measurements between CT and MR images.

For the comparison of initial computer automatically generated segmentations versus the final manually corrected results, there were 0.15% (*p* = 0.97), −0.45% (*p* = 0.93), 0.47% (*p* = 0.97), and 5.33% (*p* = 0.64) changes on the CT images, as well as −0.53% (*p* = 0.91), −1.41% (*p* = 0.81), 0.39% (*p* = 0.97), and 5.61% (*p* = 0.66) changes on the MR images, for the outer, inner, SAT, and VAT measurements, respectively. The difference between initial computer-automated versus final manual-corrected results was not statistically significant in all comparisons.

## 4. Discussion

In this study, we demonstrated there was excellent cross-modality agreement for abdominal adipose tissue quantification using the proposed computer-assisted software framework. We obtained these measurements from the same subjects who underwent CT and MR imaging on the same day. We showed the amount of SAT and VAT can be reliably measured by both imaging modalities with consistent results using this flexible software framework. The presented software provides a unified image processing platform that enables the user to quantify and compare body fat by both modalities interchangeably, along with providing a simple user interface for manual correction as well as DICOM network functions for practical workflow integration.

Several studies have compared adipose tissue measurements in the abdomen by CT and MR in animals [8] and humans [9,10,18,19,20]. Their results in general found that both modalities shared a high level of agreement for quantifying abdominal adipose tissue. However, one study found a lesser amount of visceral fat in MR compared to CT, possibly attributable to partial volume effects in early MR technology [9], and another study found a greater amount of visceral fat in MR compared to CT, perhaps attributable to the suboptimal CT thresholds used [19].

**Figure 3:**
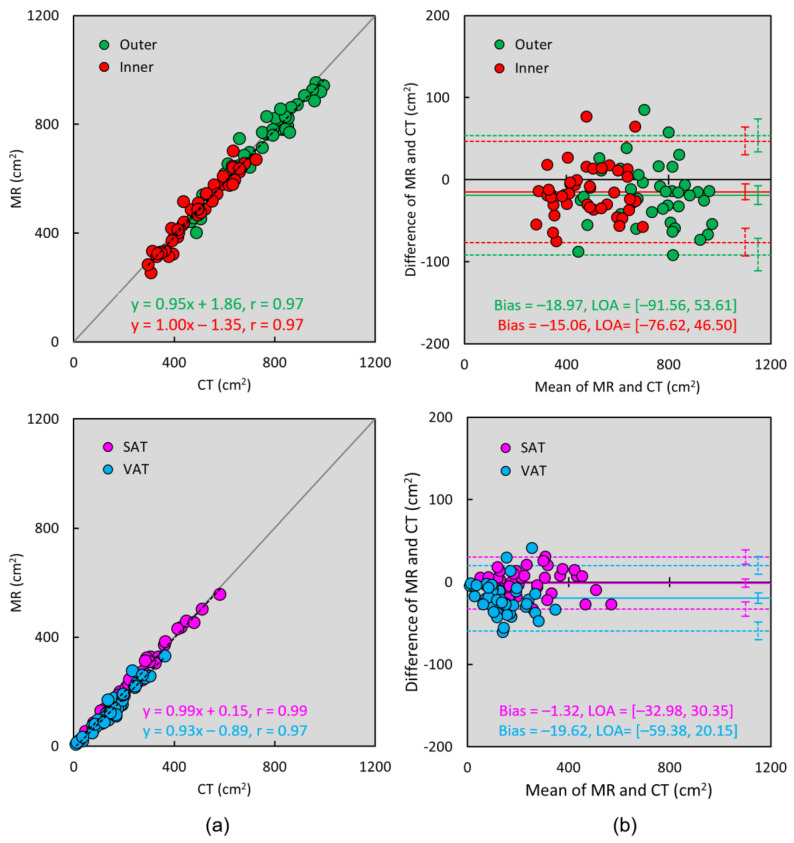
Scatter plots comparing the agreements between CT and MR image segmentation and adipose tissue quantification based on the proposed approach. (**a**) Linear regression analysis shows excellent correlations among outer (green dots) and inner (red dots) abdominal areas, as well as SAT (cyan dots) and VAT (magenta dots) measurements between CT versus MR. (**b**) Bland–Altman plots also show small variability in outer, inner, SAT, and VAT measurements between the two modalities. The horizontal color solid and dashed lines in Bland–Alman plots represent the bias and limits of agreement (LOA = mean ± 1.96 SD), and vertical lines represent the 95% confidence intervals.

In our comparisons, Table 2 showed similar results between CT and MR measurements of outer and inner abdominal regions as well as SAT and VAT. The scatter plots in Figure 3a also confirmed their good agreement. Bland–Altman plots in Figure 3b showed small variability in outer, inner, SAT, and VAT measurements between CT and MR. For our results overall, CT measurements had slightly but non-significantly higher values than the MR, particularly for the VAT.

In the literature, most of the methodologies developed for abdominal fat quantification have been designed to work with either CT or MR imaging but not both modalities. For example, Kim et al., [21], Takahashi et al. [22], Parikh et al. [23], and Ozola-Zālīte et al. [24] presented dedicated methods for abdominal fat quantification, but they only worked for CT images. Heckman et al. [19], Positano et al. [25], Liou et al. [26], Zhou et al. [27], Würslin et al. [28], and Kullberg et al. [29] developed different techniques for quantifying abdominal adipose tissues only for the MR images.

Several commercial software platforms have been used to quantify abdominal fat in CT and MR scans, but their analysis workflow required manual delineation of the abdominal wall muscle layer. These include SliceOmatic (TomoVision, Montreal, Canada) for CT analysis [24,30,31] and MR analysis [32,33], Aquarius (TeraRecon, San Mateo, CA, USA) for CT analysis [11], Analyze (AnalyzeDirect, Overland Park, KS, USA) for CT and MR analysis [10,33], and EasyVision (Philips Medical Systems, Bothell, WA, USA) for MR analysis [33]. A public domain software ImageJ (National Institutes of Health, Bethesda, MD, USA) has also been used interactively for assessing abdominal fat in MR [34] and in both CT and MR [20].

Most of the aforementioned approaches used histogram-based thresholding to differentiate adipose tissue from muscle, bone, and background regions. Similar to our framework, the majority of these software defined a preset range of pixel intensity thresholds on the CT images [20,21,22,23,24] or a default threshold value on the MR image [9,20,25,26,30] to classify fat tissues. It is worth noting that these threshold values might have been adjusted within the software and were often varied among different studies. Fuzzy c-means clustering (FCM) [35] is an alternative method to intensity thresholding for unsupervised pixel classification. FCM-based approach was also used in several studies for abdominal fat quantification [13,14,25,28,34].

These intensity-based thresholding techniques require another step to detect abdominal wall layers for differentiating between SAT and VAT for fat distribution analysis. As in our implementation, active contouring or snake-based technique was used previously to extract these abdominal body contours and define tissue boundaries [13,14,25,28,33]. After the body contour detection and fat pixel classification, the results were then visually examined by experienced readers, and corrections were made to improve the final results.

In our analysis of CT images, a HU threshold range from −190 to −45 was selected to measure SAT and VAT that produced consistent results with MRI quantification by a 50% of the maximum intensity threshold. While a HU range from −190 to −30 was more commonly used for identifying adipose tissue in CT [20,21,22,23,24], a HU range from −190 to −50 HU was found to produce excellent reproducibility and repeatability for CT-based abdominal SAT and VAT quantification [35]. Other HU thresholds have also been used on the CT images—for example, from −150 to −30 and from −205 to −51 to measure abdominal visceral fat [19,36], from −140 to −30 for measuring adipose tissue in a PET/CT system [37], and from −190 to −40 and from −190 to −45 to measure epicardial adipose tissue [38]. Therefore, there is no standard CT threshold range for measuring adipose tissue, and these thresholds may need to be adjusted and optimized for different imaging applications.

Recent developments in supervised machine learning have also shown promising results for both abdominal CT and MR fat quantification [39]. An area of particular interest is the deep learning-based approach with convolutional neural network for assessment of abdominal adipose tissue distribution by CT and MRI. The development of these newer image processing models can be very useful for generating reliable fat segmentation based on deep learning algorithms, speeding up volumetric fat quantification for the whole image scan [40], or potentially extending abdominal fat and muscle quantification for a more comprehensive body composition analysis [41,42,43]. However, none of these deep learning-based approaches were shown to work on multi-modality imaging studies, and they would require different labelled dataset to retrain new models for out-of-domain applications.

In this work, we compared abdominal fat quantification from 2D axial cross-section CT and MR images of the same subjects. Previous studies have shown that adipose tissue area quantified from a single-slice image sampled at various intervertebral disc levels had strong correlations with total abdominal and visceral adipose tissue volume among various clinical applications [44,45,46,47]. For this reason, our comparison was only focused on the level of L2–L3 and L4–L5 interspaces instead of processing the entire 3D image stack. However, this framework may also be used to quantify abdominal fat volume in a 3D image stack on a slice-by-slice basis.

There are some potential limitations in our work. The matching of different slice locations between MR and CT images may be discrepant due to tissue deformation as well as slightly varied transverse planes among two different scans. Discrepancies between CT and MR results may also come from the shape variation of the bowel during peristalsis and from different amount of food materials presented in the digestive tract at different imaging times. We have not performed an analysis of the time required for this computer-assisted approach versus manual methods, but our results suggest that the proposed framework could offer significant time savings for routine clinical application and workflow improvement. Intra- and inter-operator variability were not assessed in the current study. However, this limitation is mitigated as the operator only needs to remove false positive pixels based on the computer-generated masks. Finally, there is a need to evaluate the software on a larger population in future studies.

## 5. Conclusions

We showed that the two main abdominal adipose tissues (VAT and SAT) can be reliably quantified from both CT and Dixon MR images of the same subjects with excellent agreement using a unified computer-assisted software framework. This flexible image processing platform offers a simple-to-use workflow to facilitate consistent abdominal wall segmentation and adipose tissue quantification for both imaging modalities. The presented framework will be applied to support various clinical research applications for routine abdominal fat quantification.

## Figures and Tables

**Table 1:** Demographic information for 21 study participants undergoing same day abdominal CT and MR Imaging.

Sex	Male: 12 (57%); Female: 9 (43%)			
	Mean	SD	Max	Min
Age (year)	56	14	78	31
Height (cm)	169.7	10.6	190.0	155.0
Weight (kg)	79.1	14.3	100.0	51.0
Body Mass Index (kg/m^2^)	27.5	4.6	37.9	18.7

**Table 2:** Summary comparison of abdominal regions and adipose tissues measured from 42 matched CT and MR images.

**CT**	**Mean**	**SD**	**Max**	**Min**
Outer (cm^2^)	736.3	163.2	996.2	400.3
Inner (cm^2^)	489.3	121.2	727.2	297.1
SAT (cm^2^)	242.1	127.2	582.5	46.9
VAT (cm^2^)	149.4	81.2	363.5	9.5
**MR**	**Mean**	**SD**	**Max**	**Min**
Outer (cm^2^)	717.3	159.0	952.4	382.3
Inner (cm^2^)	474.2	124.8	701.4	253.6
SAT (cm^2^)	240.7	126.7	555.7	52.8
VAT (cm^2^)	129.8	77.8	330.4	5.6

## Data Availability

Not applicable.

## Abbreviations

CT	computed tomography
DICOM	digital imaging and communications in medicine
HU	Hounsfield unit
MRI	magnetic resonance imaging
PACS	picture archiving and communication system
SAT	subcutaneous adipose tissue
VAT	visceral adipose tissue
